# Primary umbilical malignant melanoma requiring full-thickness abdominal wall resection and reconstruction with autologous fascia lata: a case report

**DOI:** 10.1080/23320885.2025.2594830

**Published:** 2025-11-24

**Authors:** Yasue Kurokawa, Yoshihiro Sowa, Soichiro Kado, Yuki Kimura, Takeo Maekawa, Mayumi Komine, Kotaro Yoshimura

**Affiliations:** ^a^ Department of Plastic Surgery, Jichi Medical University Hospital, Shimotsuke-shi, Tochigi, Japan; ^b^ Department of Dermatology, Jichi Medical University Hospital, Shimotsuke-shi, Tochigi, Japan; ^c^ Department of Gastroenterological Surgery, Jichi Medical University Hospital, Shimotsuke-shi, Tochigi, Japan; ^d^ Department of Dermatology, Jichi Medical University Saitama Medical Center, Saitama-shi, Saitama, Japan

**Keywords:** Abdominal wall, fascia lata, malignant melanoma, reconstructive surgical procedures, umbilicus

## Abstract

Primary malignant melanoma of the umbilicus is extremely rare; evidence guiding optimal resection and reconstruction is limited. A 47-year-old man with primary umbilical melanoma underwent full-thickness abdominal wall resection including the peritoneum. The abdominal wall defect was reconstructed using an autologous fascia lata graft. Histopathology confirmed malignant melanoma with a tumor thickness of 18 mm (pT4bN1aM0, Stage IIIC). At 6-month follow-up, no local recurrence or incisional hernia was observed, and at one year after surgery, no evidence of herniation or recurrence was noted. Autologous fascia lata offers a practical option for abdominal wall reconstruction after extensive oncologic resection of the umbilical region, achieving early freedom from recurrence and hernia in this case.

## Introduction

Primary malignant melanoma arising in the umbilical region is a rare clinical entity. Radical excision is the mainstay of treatment, often requiring complex abdominal wall reconstruction. However, literature regarding the appropriate extent of resection and reconstructive strategies remains limited. Herein, we report a rare case of primary umbilical melanoma requiring full-thickness resection including the peritoneum, followed by abdominal wall reconstruction using autologous fascia lata, with a brief review of the literature.

## Case report

A 47-year-old man presented to a previous hospital with a gradually enlarging black macule in the umbilical region. He had noticed the lesion approximately one year earlier, which later developed into a palpable mass. An incisional biopsy confirmed a malignant melanoma with ulceration and Breslow thickness exceeding 4 mm. He was referred to our dermatology department for further evaluation in June 2024. He had no significant medical, family, or smoking history.

**Figure 1.**
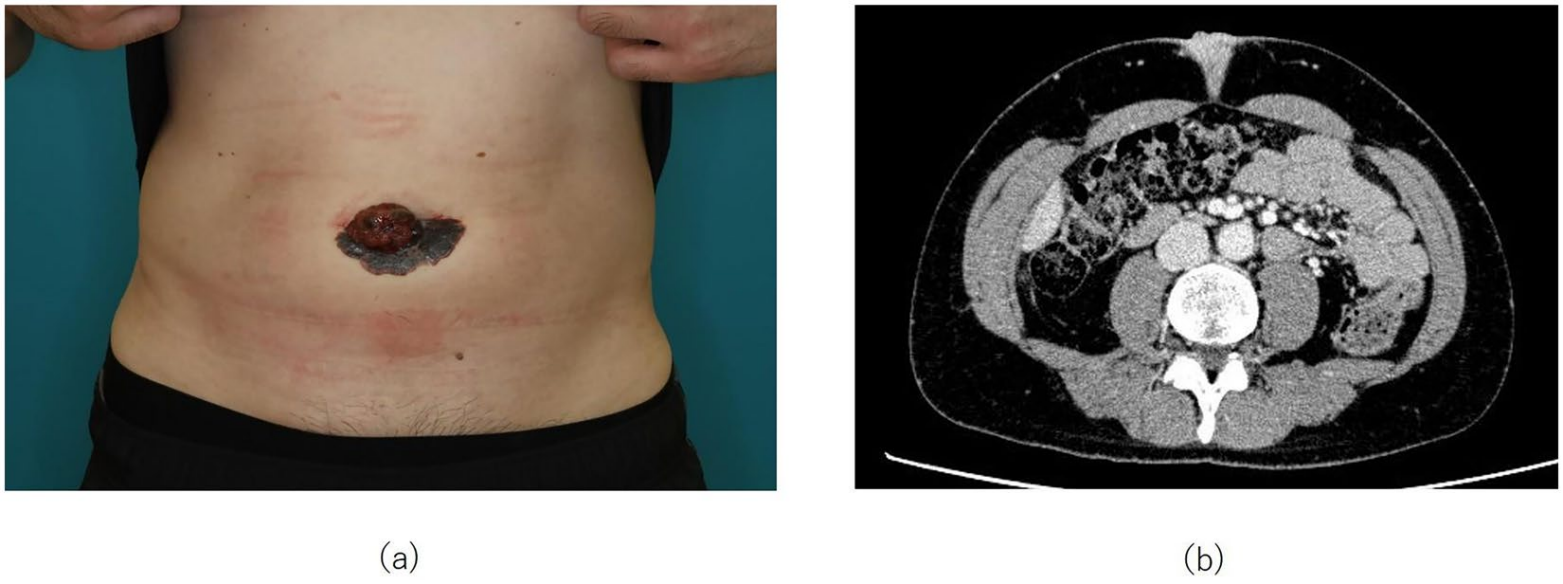
Initial presentation at our department. (a) Clinical photograph. A 67 × 32 mm black macule is observed at the umbilicus, with a 38 × 22 mm reddish nodule centrally located within the lesion. (b) Contrast-enhanced axial CT image. The tumor shows invasion extending to the fascia.

At the initial presentation, physical examination revealed a 67 × 32 mm black macule with a central mass in the umbilical region with a central 38 × 22 mm erythematous nodule (Figure 1a). Whole-body computed tomography (CT) showed no obvious lymph node or visceral metastases (Figure 1b). The clinical diagnosis was cT4bN0M0, Stage IIC, and the treatment plan included primary tumor resection and sentinel lymph node biopsy. The extent of resection was determined through a preoperative conference involving dermatologists, gastrointestinal surgeons, and plastic surgeons. In accordance with the NCCN Guidelines (National Comprehensive Cancer Network), a 2-cm lateral margin was planned, and the deep margin was set to include the underlying fascia. A literature search regarding the appropriate extent of resection for primary umbilical malignant melanoma revealed reports recommending combined resection including the peritoneum [1–4]. Based on these reports, a surgical plan was established to resect the tumor with a 2-cm lateral margin, including the anterior rectus sheath and the peritoneum.

**Figure 2.**
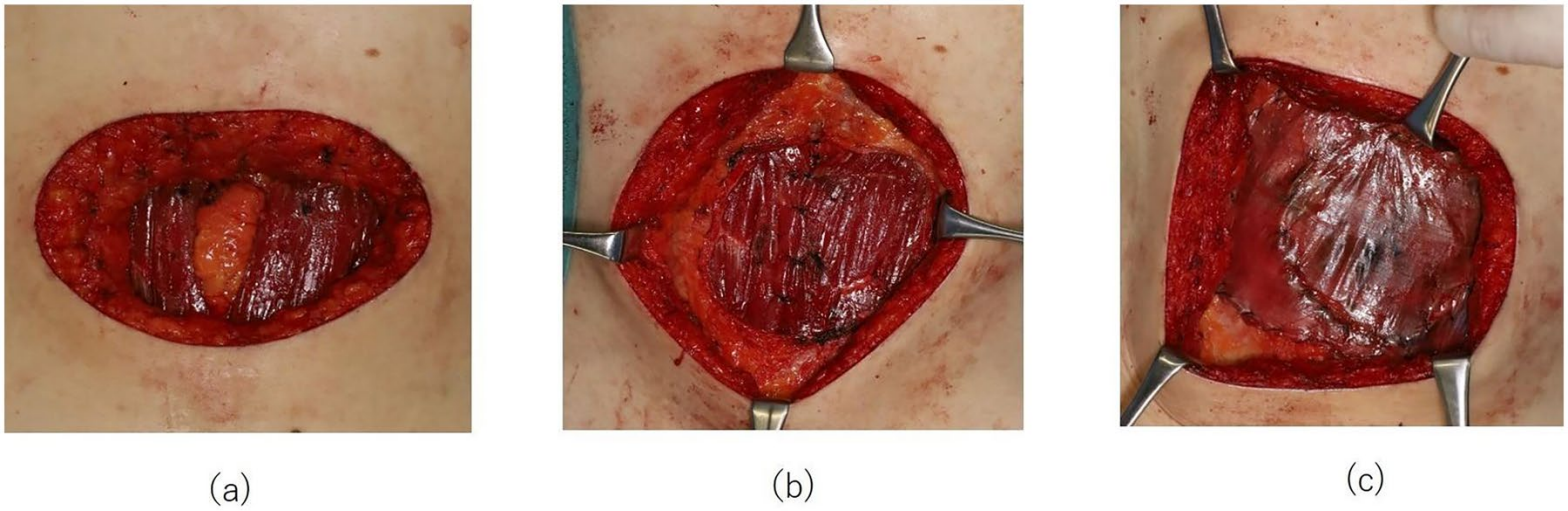
Intraoperative findings. (a) Post-resection defect following tumor excision. (b) After suturing of the rectus abdominis muscles. Due to tension during closure, the rectus abdominis muscles were approximated and sutured together to reinforce the repair. (c) After fixation of the fascia lata graft.

Surgery was performed in June 2024 under general anesthesia. The primary tumor was resected including the umbilicus, with a 2-cm lateral margin taken from the skin lesion. The resection was performed with the subcutaneous adipose tissue and the anterior rectus sheath attached to the tumor side (Figure 2a). The resection was extended from the linea alba to the peritoneum in continuity, ensuring the peritoneum remained on the tumor side. After suturing the peritoneum, the posterior rectus sheath was also closed. However, the closure was performed under considerable tension, and the central portion could not be approximated. In addition, a 10 × 7 cm defect in the anterior rectus sheath was noted (Figure 2b), and reconstruction with fascia lata was planned. First, the bilateral rectus abdominis muscles were approximated with absorbable sutures. Then, a 12 × 9 cm segment of fascia lata harvested from the right lateral thigh was placed over the rectus muscles, trimmed to be slightly larger than the anterior sheath defect, and sutured to the edges of the anterior sheath. Subsequently, the graft was circumferentially secured to the anterior sheath using continuous sutures with barbed absorbable suture (1-0 STRATAFIX^®^; Ethicon, USA), thereby reconstructing the anterior rectus sheath (Figure 2c). Skin closure was performed by approximation without tension, and the operation was completed. Postoperatively, the patient was instructed to wear an abdominal binder for approximately one month to avoid increasing intra-abdominal pressure. At the time of tumor resection, sentinel lymph node biopsy of the right inguinal region was also performed.

Histopathological examination confirmed malignant melanoma, with a tumor thickness of 18 mm, corresponding to a diagnosis of pT4b. Metastasis to the right inguinal lymph node was identified, resulting in a diagnosis of pN1a.

Molecular testing revealed a BRAF V600E mutation. In accordance with current guidelines, adjuvant immunotherapy with pembrolizumab (anti–PD-1 antibody) was initiated after surgery. At four months postoperatively, pulmonary metastases were detected, and systemic therapy was therefore intensified to combined immunotherapy with nivolumab and ipilimumab.

**Figure 3.**
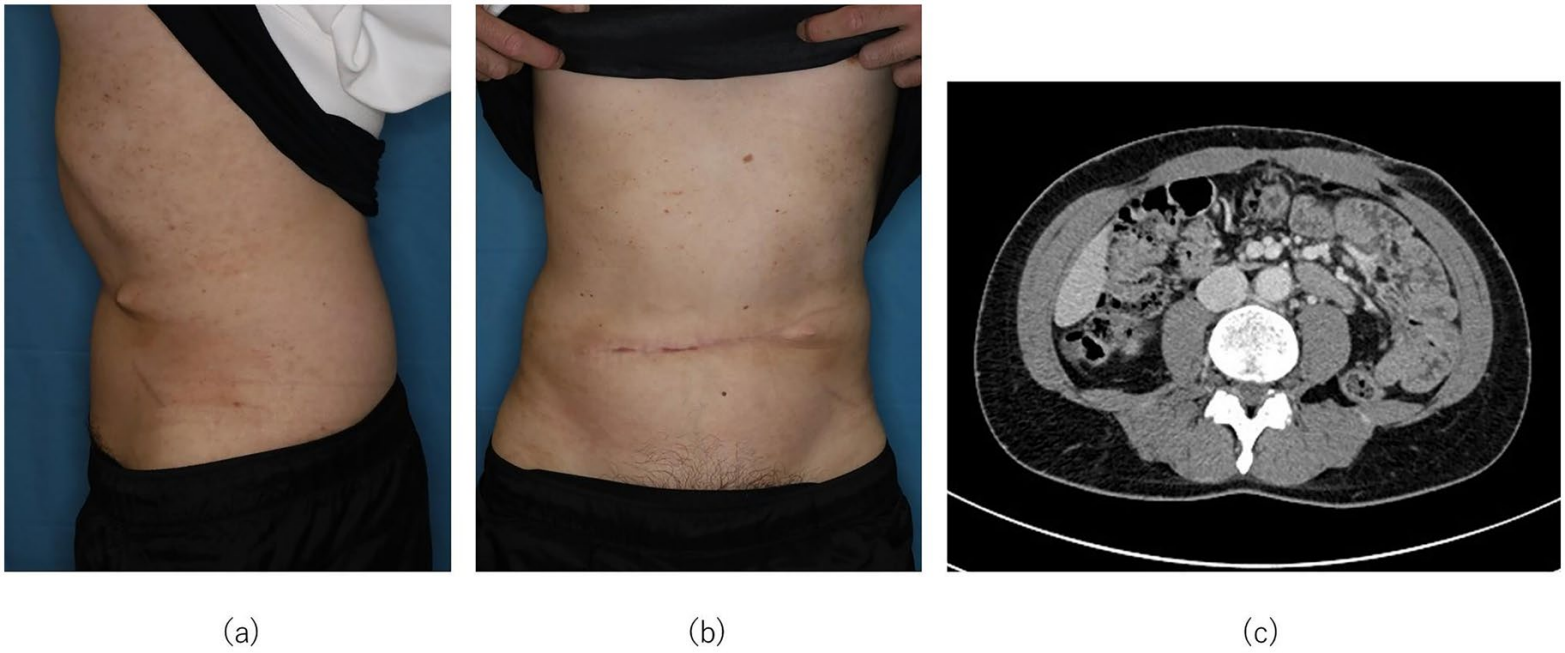
Six months postoperatively. (a) Lateral view showing a flat abdominal contour without evidence of hernia. (b) Frontal postoperative photograph demonstrating no bulging at the umbilical site. (c) No evidence of hernia was observed on follow-up CT imaging.

As of six months postoperatively, treatment is ongoing. However, the abdominal contour has remained flat with no evidence of hernia (Figure 3a), and a frontal postoperative photograph shows no bulging at the umbilical site (Figure 3b). Computed tomography has revealed no obvious local recurrence at the umbilical site (Figure 3c). At one year after surgery, no evidence of herniation or recurrence was observed.

## Discussion

Primary malignant melanoma of the umbilicus is exceptionally rare; to date only 46 cases have been reported and, to our knowledge, no detailed descriptions of abdominal-wall reconstruction techniques have been published [5]. This case therefore offers the first comprehensive account of how to manage the sizable fascial defect that follows oncologically appropriate resection.

Based on prior reports, wide composite resection including the peritoneum is generally recommended [1–4]. This approach is anatomically reasoned: the umbilicus maintains midline continuity with the parietal peritoneum through embryologic remnants such as the urachus, and incomplete excision may increase the risk of local recurrence or intraperitoneal spread. Consistent with this rationale, all four prior reports recommended wide composite excision of the entire umbilical apparatus—including its peritoneal attachment [1–4].

Such radical excision inevitably creates a central midline fascial defect. In our patient, the tumor measured 67 mm—among the largest documented—resulting in a large 10 × 7 cm defect of the anterior rectus sheath. Because the resection included the peritoneum, additional defects were also created in the peritoneum and the posterior rectus sheath. The peritoneum was closed primarily without undue tension, whereas closure of the posterior rectus sheath required considerable tension and the central portion could not be approximated.

Failure to reinforce large midline defects predisposes to early herniation. Suzuki et al. reported acute hernia the day after primary fascial closure in a similar melanoma, necessitating emergent mesh repair [4]. Taken together with the size of our defect, this experience led us to regard prophylactic reinforcement of the anterior sheath as essential.

The choice of reinforcement material requires balancing mechanical strength with long-term safety. Prosthetic mesh provides high tensile strength and low hernia recurrence but carries a non-negligible risk of infection, chronic pain, and other mesh-related complications on long-term follow-up [6]. These concerns are especially relevant in patients anticipating systemic therapy, such as immunotherapy or targeted therapy and—in women—future pregnancy, where mesh has been associated with persistent abdominal pain and even vacuum-assisted delivery attributable to impaired Valsalva manoeuvre [7,8]. Although our patient was male, these considerations underscore that autologous fascia may be preferable in women of childbearing potential.

In our case, although the posterior rectus sheath could be approximated partially under tension, we did not place a prosthetic mesh in the retro-rectus space. The decision was made in consideration of the anticipated need for systemic therapy, as prosthetic material would increase the risk of infection and mesh-related complications. Instead, we selected autologous fascia lata as an onlay reinforcement of the anterior sheath, which minimized infection risk while providing sufficient mechanical support.

In addition to material selection, reconstructive options such as local flaps have also been reported. While local flaps can be useful for extensive skin or subcutaneous tissue defects, several limitations should be noted. Lam et al. reported two cases reconstructed with propeller flaps, both complicated by postoperative hernias and long abdominal incisions including donor-site scars [9]. Brunetti et al. described pedicled perforator flaps, but flap necrosis occurred in one case requiring secondary reconstruction [10]. Thus, although local flaps may provide cosmetic advantages such as shorter scars, they do not reinforce the anterior rectus sheath and may increase the risk of herniation; furthermore, flap failure requiring reoperation would be especially undesirable in patients awaiting systemic therapy. In this context, direct closure supplemented by fascial reinforcement appears more reliable, with dog-ear deformities representing a minor issue easily corrected under local anesthesia.

Technical execution is critical to the success of autologous reconstruction. We used an onlay technique analogous to the Lichtenstein repair: the graft was trimmed approximately 1 cm larger than the defect and secured to the anterior rectus sheath with circumferential, two-layer fixation. Barbed absorbable sutures were chosen because they exhibit higher tensile strength and superior tissue-holding capacity than conventional smooth sutures, features advantageous in high-tension closures [11].

In summary, wide composite resection combined with onlay autologous fascia lata reinforcement produced a durable abdominal wall without hernia at six-month follow-up. We propose a pragmatic algorithm for this rare malignancy: (i) wide composite excision of the entire umbilical apparatus, including its peritoneal attachment; (ii) routine fascial reinforcement for large midline defects; and (iii) preference for autologous tissue when postoperative immunosuppression is anticipated or future pregnancy is possible. Larger case series and longer follow-up are needed to validate these recommendations.
